# Supplementary figures and images for: Starvation-induced autophagy via calcium-dependent TFEB dephosphorylation is suppressed by Shigyakusan
Source: PLoS One. 2020 Mar 5;15(3):e0230156. doi: 10.1371/journal.pone.0230156 (PMC7058311; doi:10.1371/journal.pone.0230156)

# Supplementary Fig 1

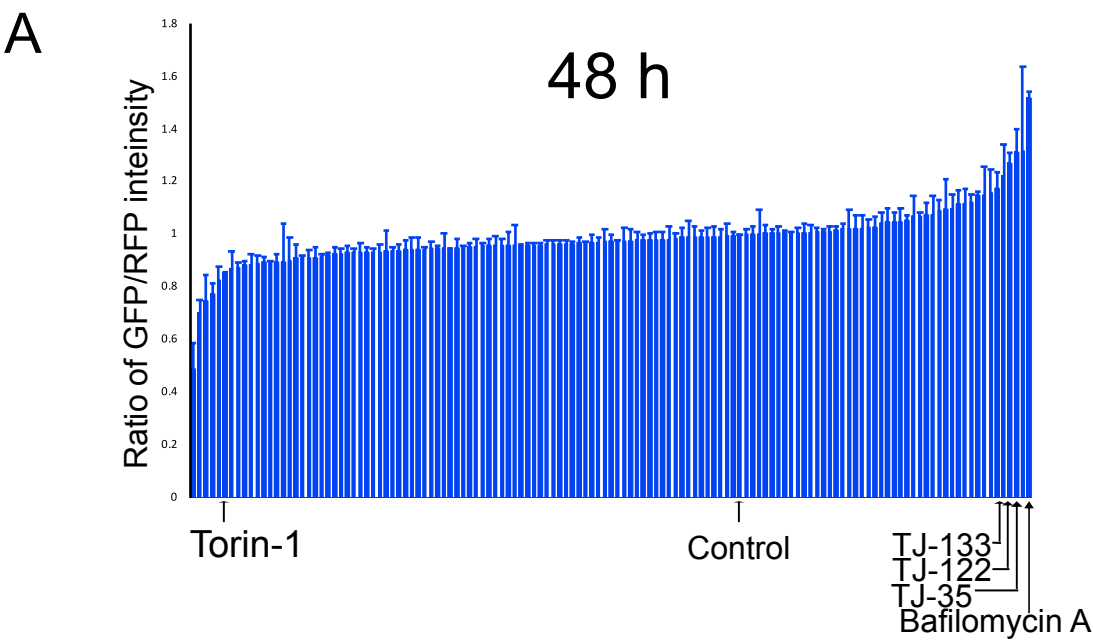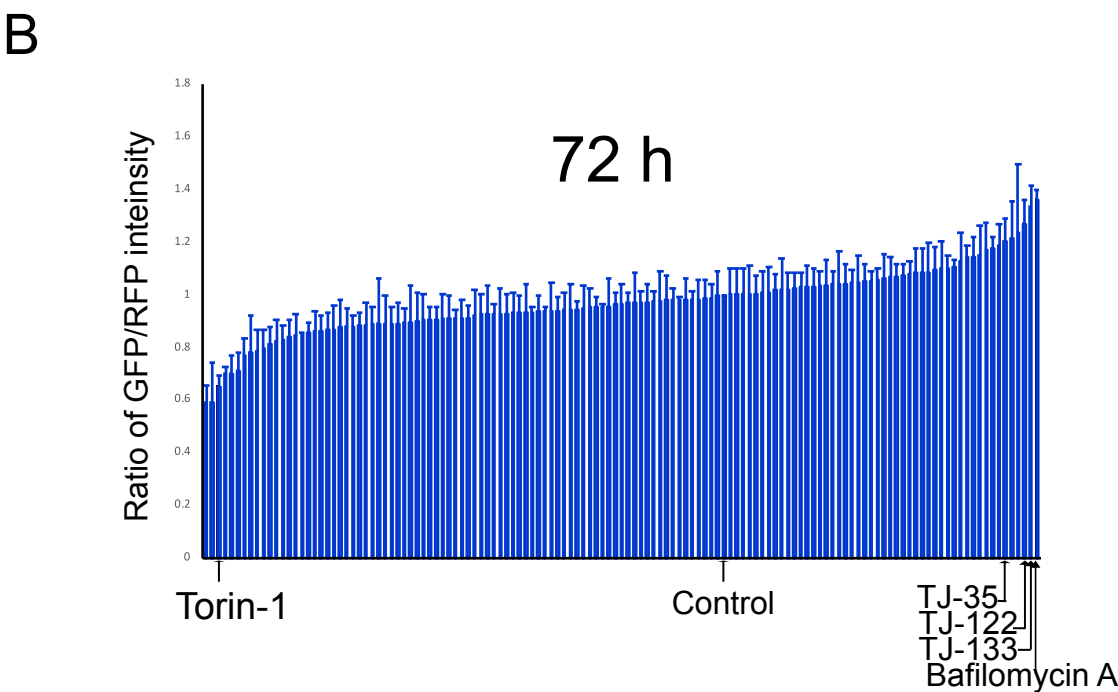

Supplement: S1 Fig — A.B. Screening results of tf-LC3 assay upon treatment with 128 Kampo medicines. The signal intensity ratio of GFP/RFP in each view field at 48 h (A) or 72 h (B) incubation is presented in order of its value. Average and standard deviation of three independent screens are shown. Bafilomycin A1 and Torin-1 were used as controls. (PDF) [file pone.0230156.s001.pdf]

Supplementary Fig 2

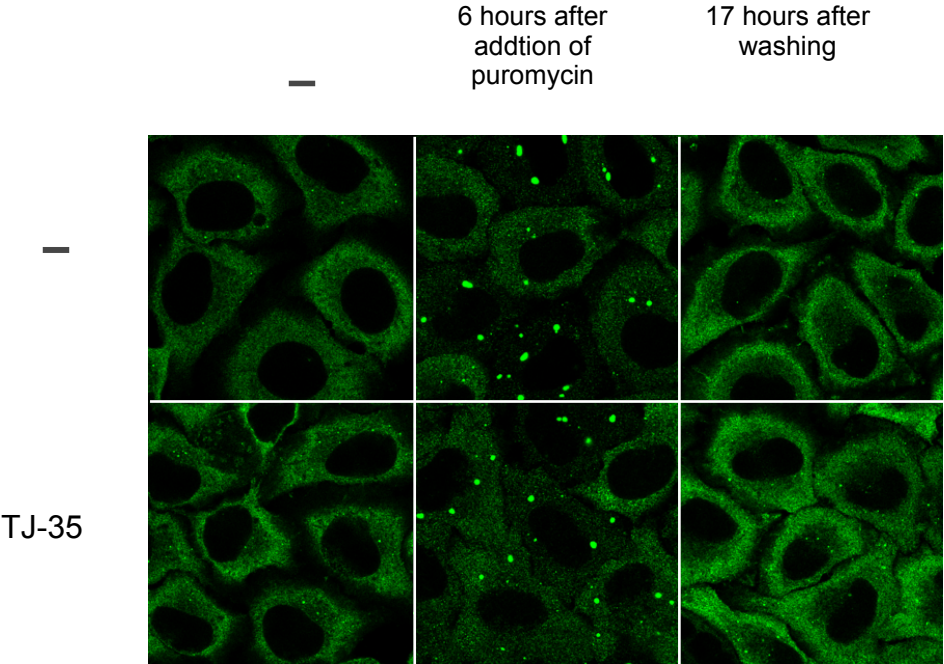

Supplement: S2 Fig — HeLa cells were cultured in DMEM with or without puromycin for 6 h, and after being washed out, further cultured in DMEM with or without TJ-35 for 17 h, and immunostained with anti-p62 on SP-8. (PDF) [file pone.0230156.s002.pdf]

# Supplementary Fig 3

A

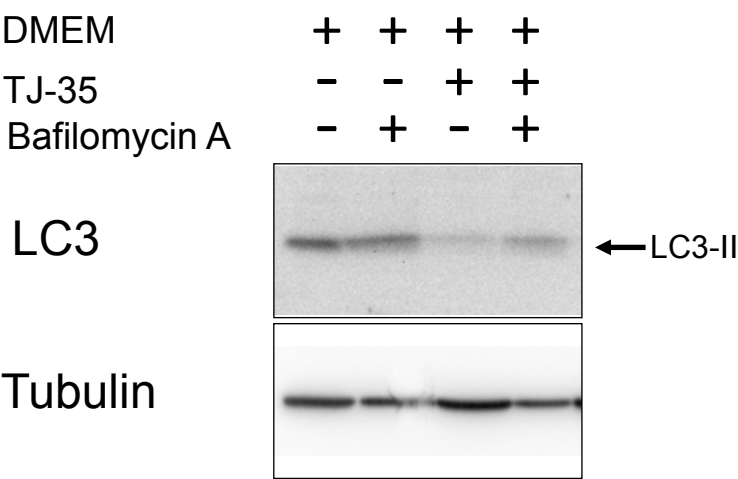

B

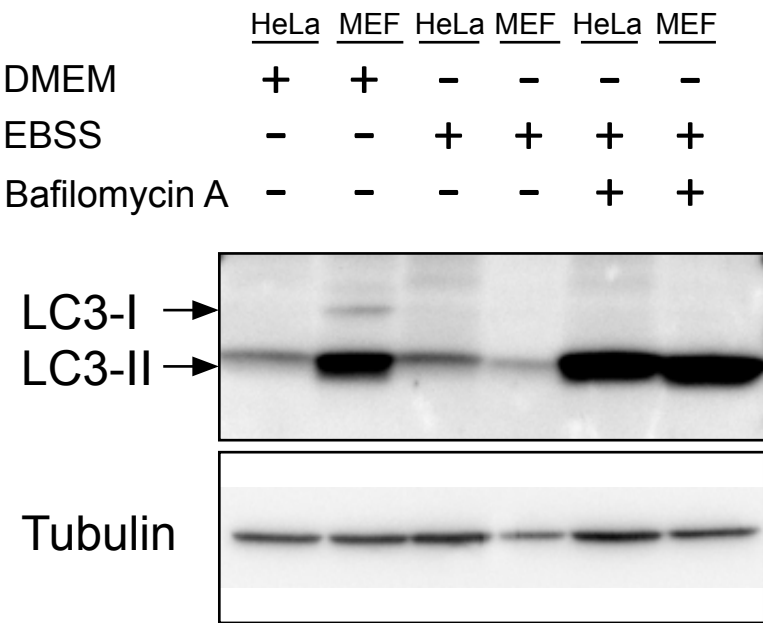

Supplement: S3 Fig — A. HeLa cells were treated with or without TJ35 in DMEM, with or without bafilomycin A1, for 4 h. The lysates were assessed by Western Blotting with LC3 antibody. B. Comparison of Band pattern of LC3 by western blotting: MEF and HeLa cells were cultured in DMEM or EBSS, with or without bafilomycin A1, for 4 h. The lysates were assessed by western blotting with antibodies against LC3 and tubulin (PDF) [file pone.0230156.s003.pdf]

# Supplementary Fig 4

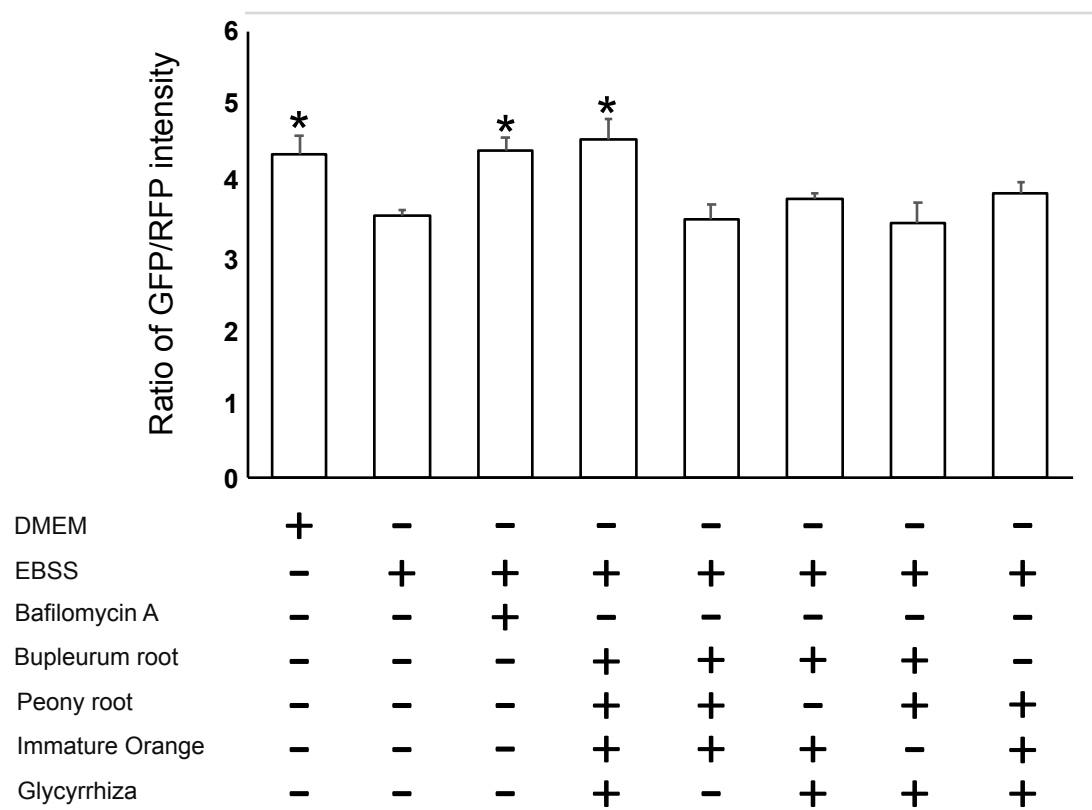

Supplement: S4 Fig — Tf-LC3–expressing HeLa cells were cultured in DMEM with or without Shigyakusan and with extracts with omission of any of the four crude drugs for 4 h, shifted to DMEM or EBSS with or without the above combination of Shigaykusan ingredients for 2 h, and observed on SP-8. The graph below shows the signal intensity ratio of GFP/RFP in each field of view. * denotes p<0.05 (unpaired two-tailed Student’s t-test) against EBSS only sample. (PDF) [file pone.0230156.s004.pdf]

Supplementary Fig 5

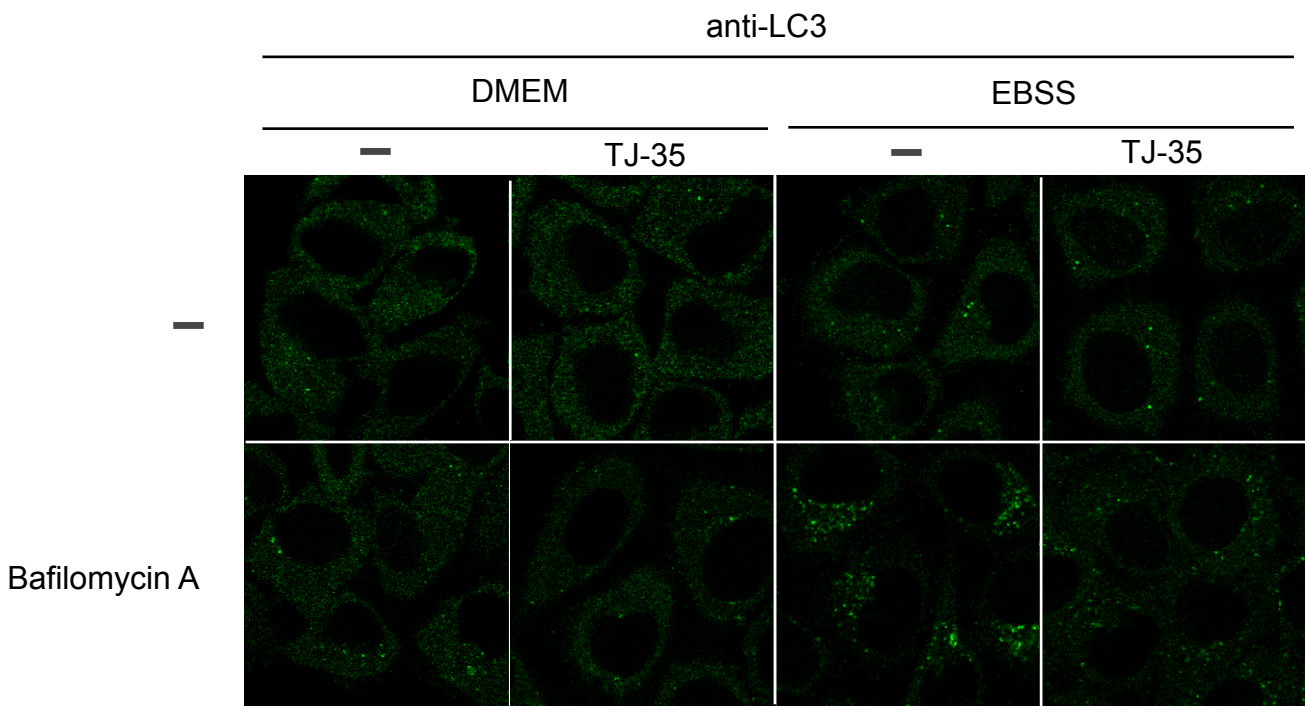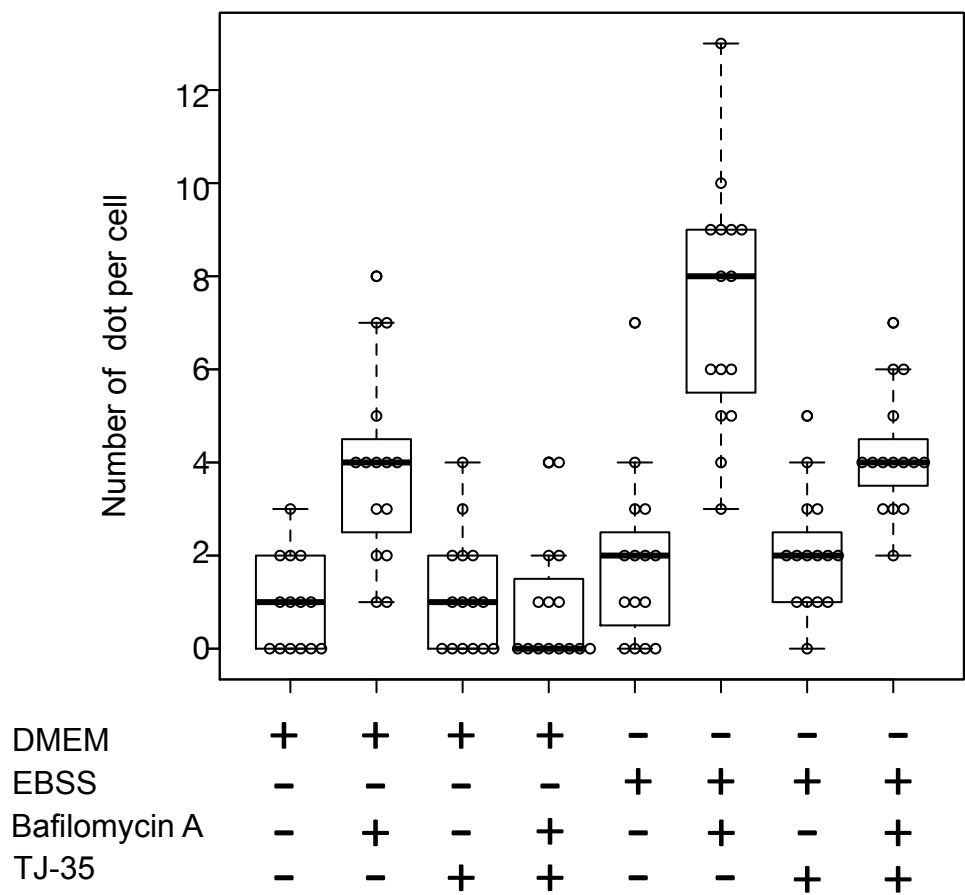

Supplement: S5 Fig — HeLa cells were treated with or without TJ35 in DMEM or EBSS, with or without bafilomycin A1, for 4 h. The cells were immunostained with anti-LC3 antibody. The graph shows Alexa Fluor 488-positive puncta per cell. Median: line; upper and lower quartiles: boxes; 1.5-interquartile range: whiskers. (PDF) [file pone.0230156.s005.pdf]

Supplementary Fig6

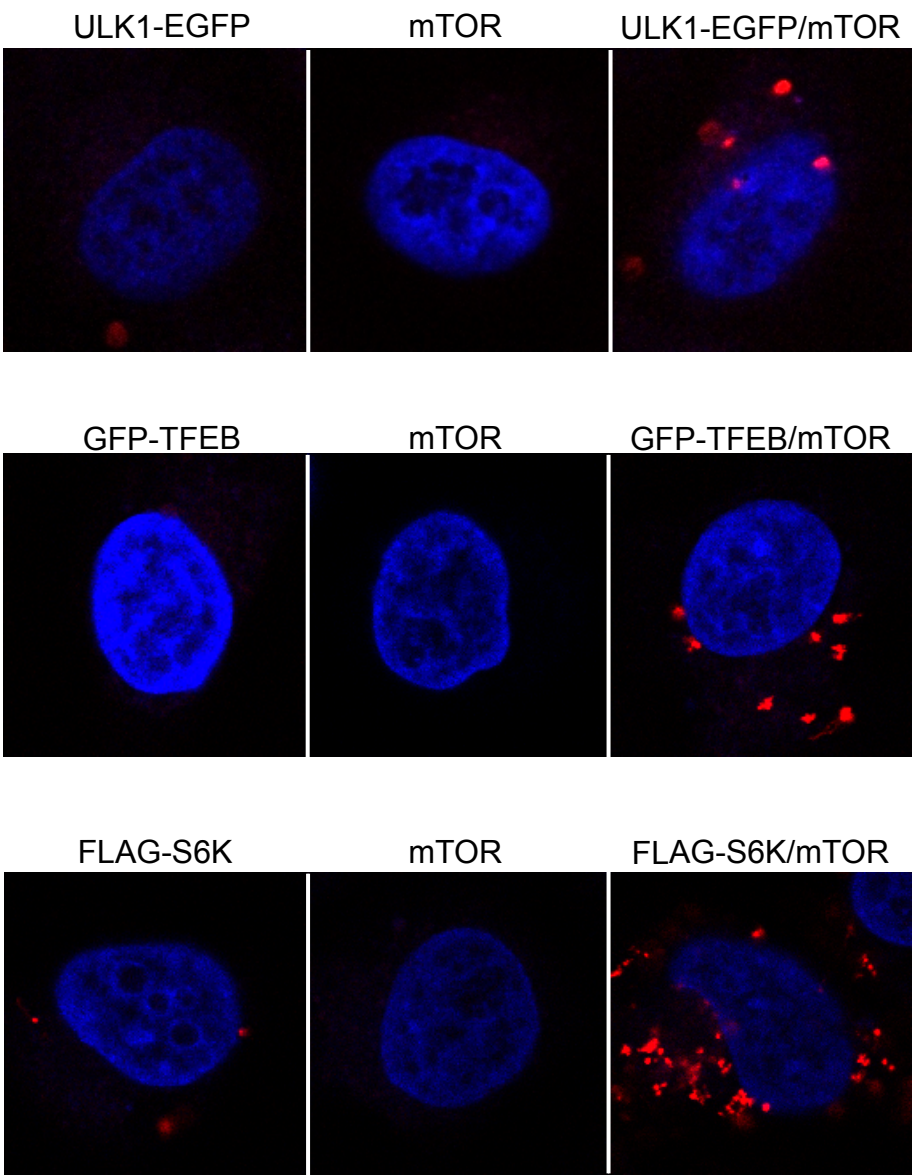

Supplement: S6 Fig — ULK1-EGFP–expressing HeLa cells and GFP-TFEB–expressing HeLa were cultured in DMEM for 24 h, and subjected to PLA using either anti-GFP antibody or mTOR antibody or both. FLAG-S6K–expressing HeLa were cultured in DMEM for 24 h, and subjected to PLA using either anti-FLAG antibody or mTOR antibody or both. (PDF) [file pone.0230156.s006.pdf]

Supplementary Fig 7

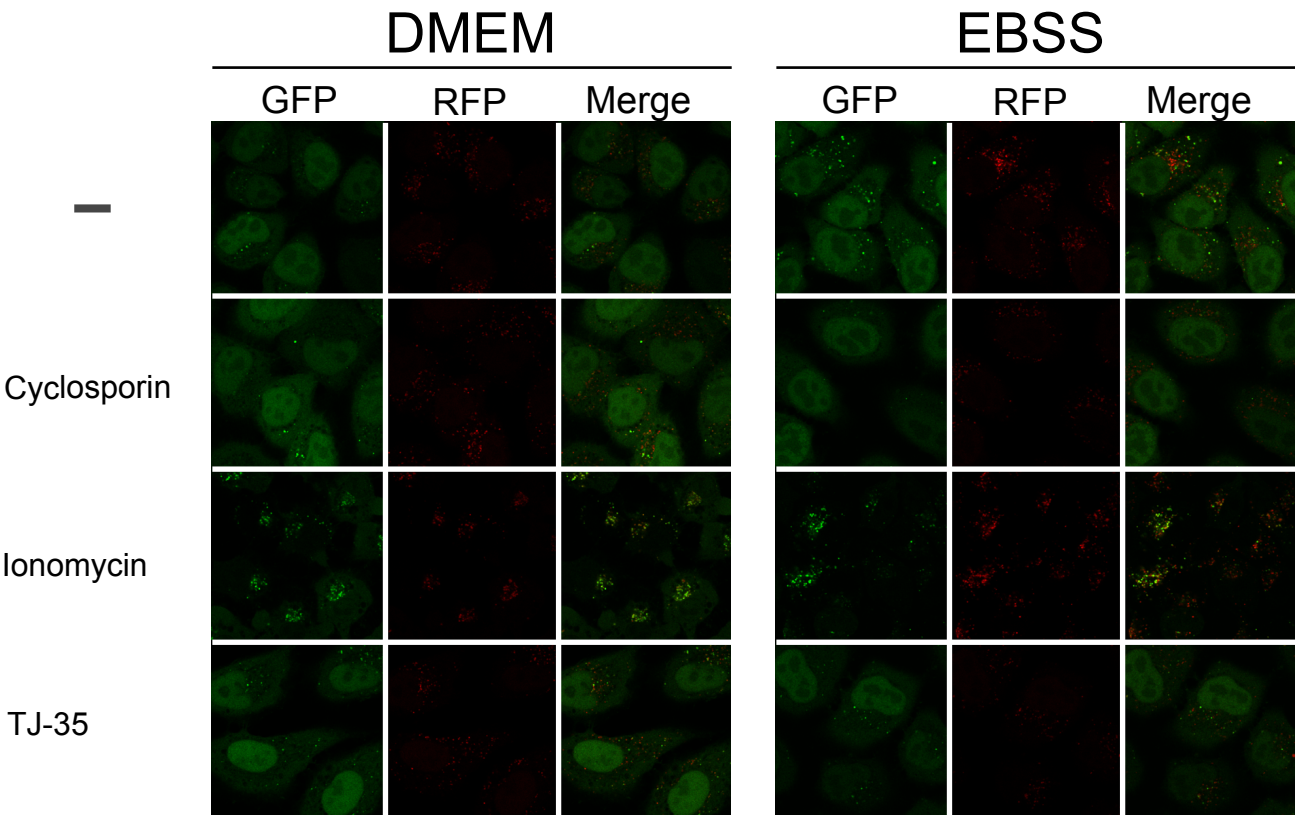

Supplement: S7 Fig — Tf-LC3–expressing HeLa cells were treated in DMEM or EBSS with 3 μM ionomycin or 20 μM cyclosporin A for 30 min. TJ-35 treatment condition was the same as above. Images were acquired on SP-8. (PDF) [file pone.0230156.s007.pdf]

Full blot images-Figure 2C

|               |   |   |   |   |
|---------------|---|---|---|---|
| EBSS          | + | + | + | + |
| TJ-35         | - | - | + | + |
| Bafilomycin A | - | + | - | + |

LC3-II

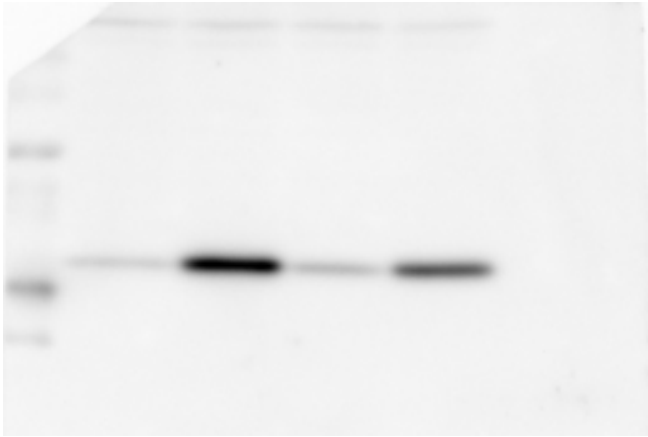

Tubulin

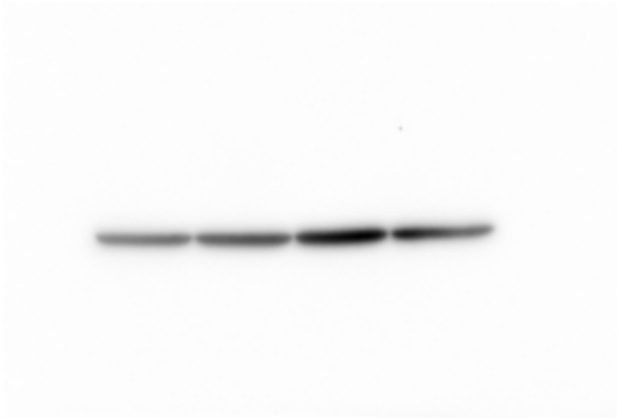

Supplement: S8 Fig — (PDF) [file pone.0230156.s008.pdf]

Full blot images-Figure 4C

C

|         |   |   |   |   |   |   |
|---------|---|---|---|---|---|---|
| EBSS    | - | + | + | - | + | + |
| DMEM    | + | - | - | + | - | - |
| TJ-35   | - | - | - | + | + | + |
| Torin-1 | - | - | + | - | - | + |

p-ULK1

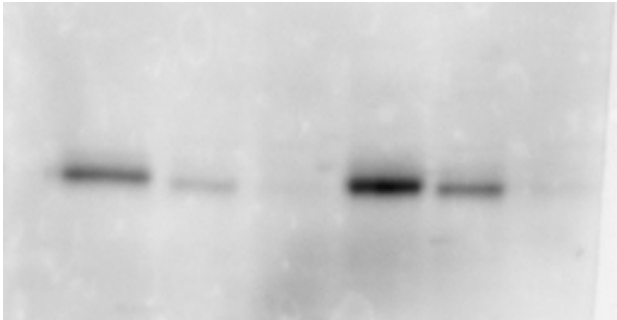

ULK1

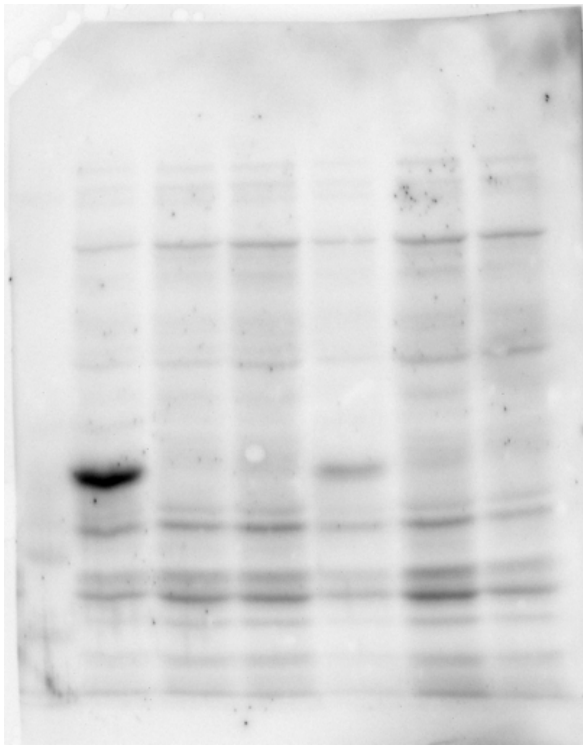

Tubuli

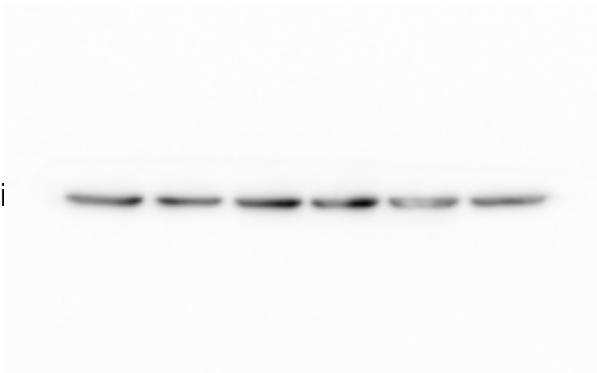

Supplement: S9 Fig — (PDF) [file pone.0230156.s009.pdf]

Full blot images-Figure 4D

D

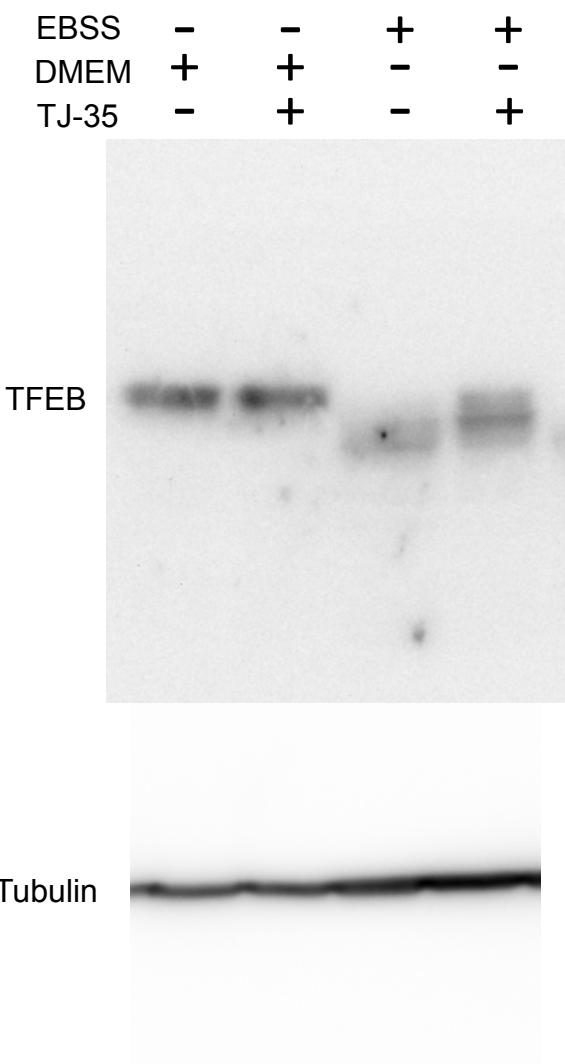

Supplement: S10 Fig — (PDF) [file pone.0230156.s010.pdf]

Full blot images-Figure 4E

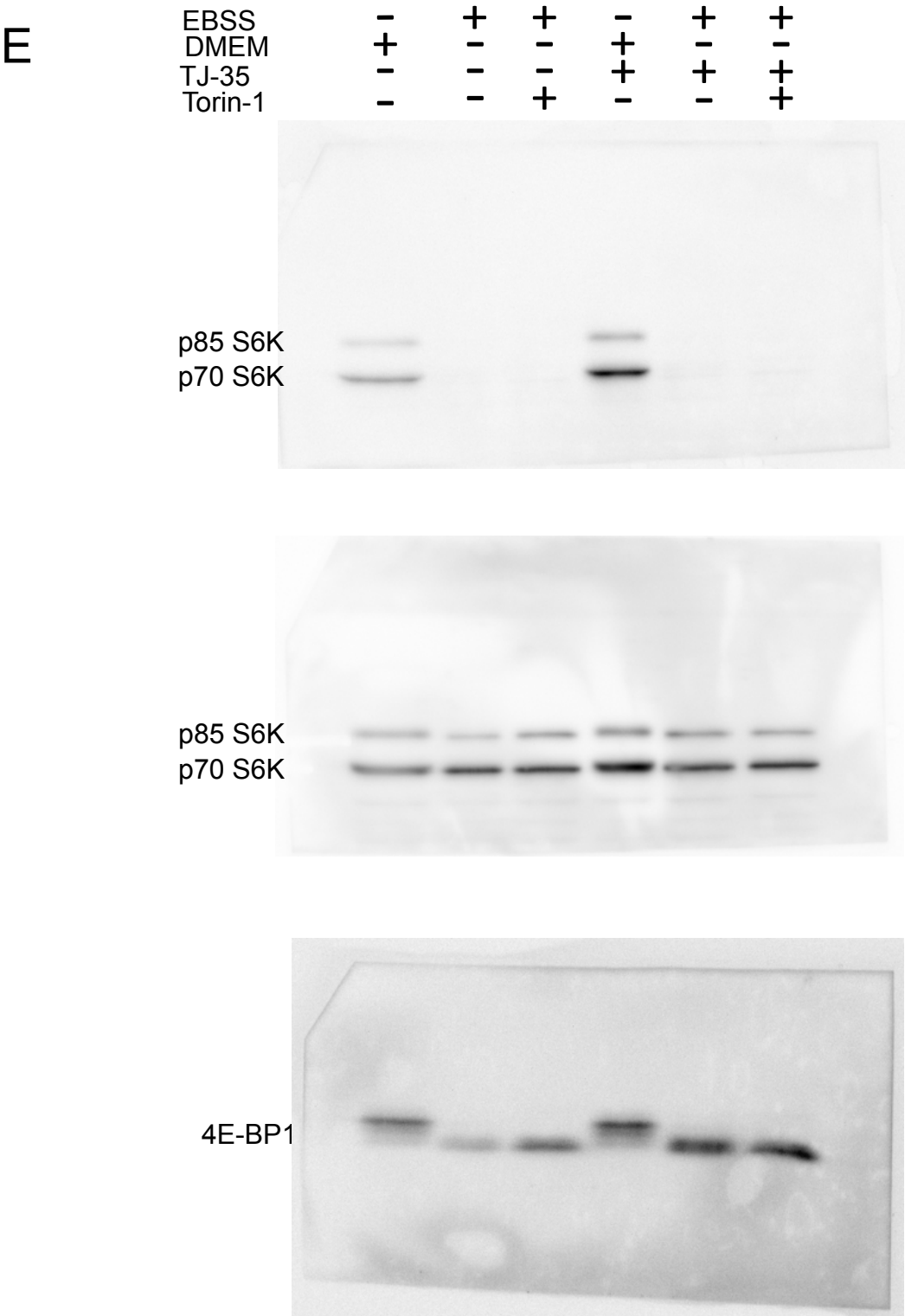

Supplement: S11 Fig — (PDF) [file pone.0230156.s011.pdf]

# Full blot images-Figure 6-1

|                    |   |   |   |    |    |
|--------------------|---|---|---|----|----|
| Ionomycin          | - | + | + | +  | +  |
| Cyclosporin A (μM) | - | - | 1 | 10 | 20 |

TFEB

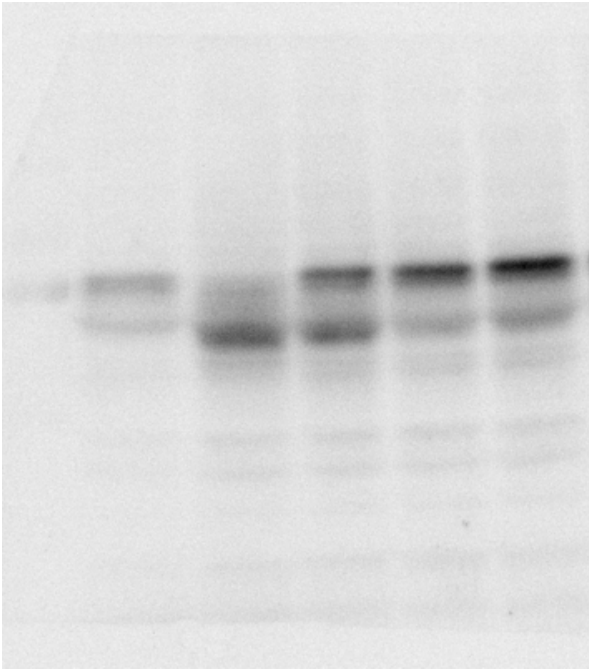

Tubulin

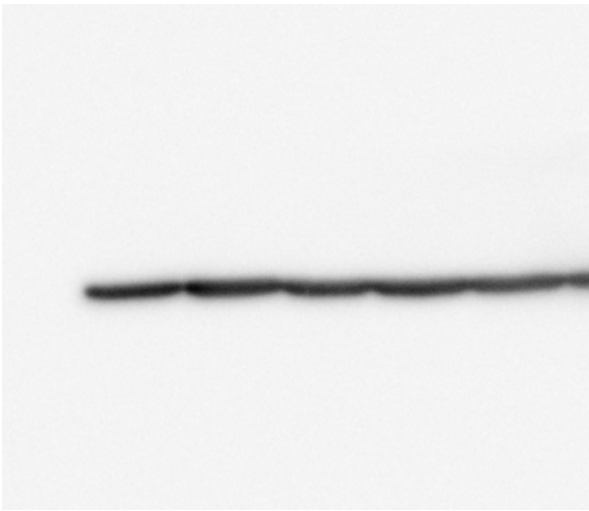

Supplement: S12 Fig — (PDF) [file pone.0230156.s012.pdf]

## Full blot images-Figure 6-2

|                          |   |   |   |    |    |
|--------------------------|---|---|---|----|----|
| Ionomycin                | - | + | + | +  | +  |
| Cyclosporin A ( $\mu$ M) | - | - | 1 | 10 | 20 |

NFAT

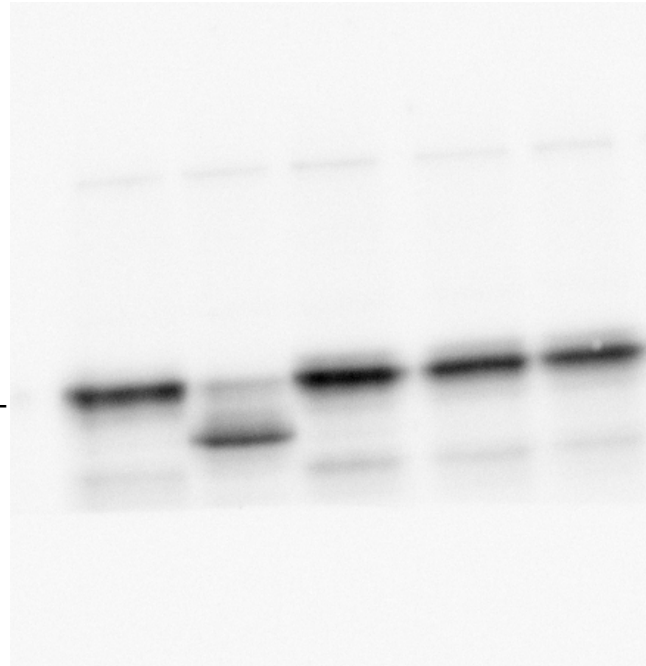

Tubulin

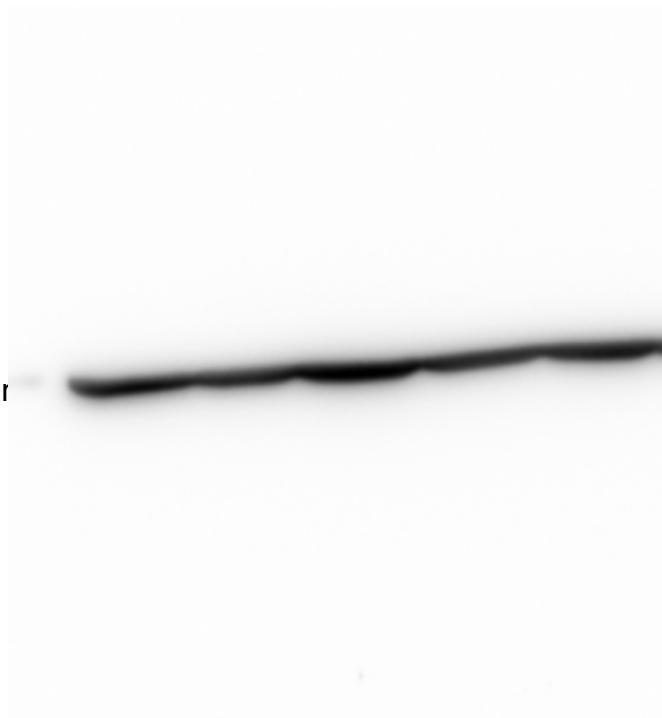

Supplement: S13 Fig — (PDF) [file pone.0230156.s013.pdf]
