# Supplementary material for: Starvation-induced autophagy via calcium-dependent TFEB dephosphorylation is suppressed by Shigyakusan
Source: PLoS One. 2020 Mar 5;15(3):e0230156. doi: 10.1371/journal.pone.0230156 (PMC7058311; doi:10.1371/journal.pone.0230156)

Full blot images-Supplementary Fig 3

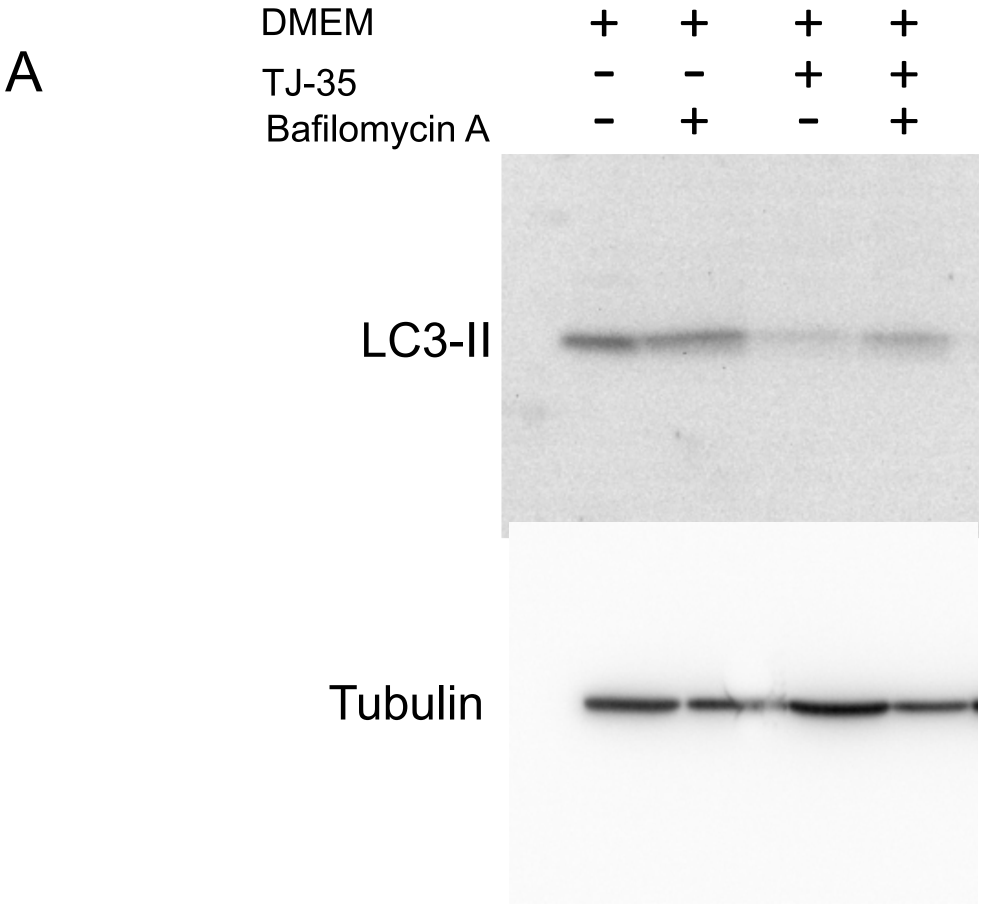

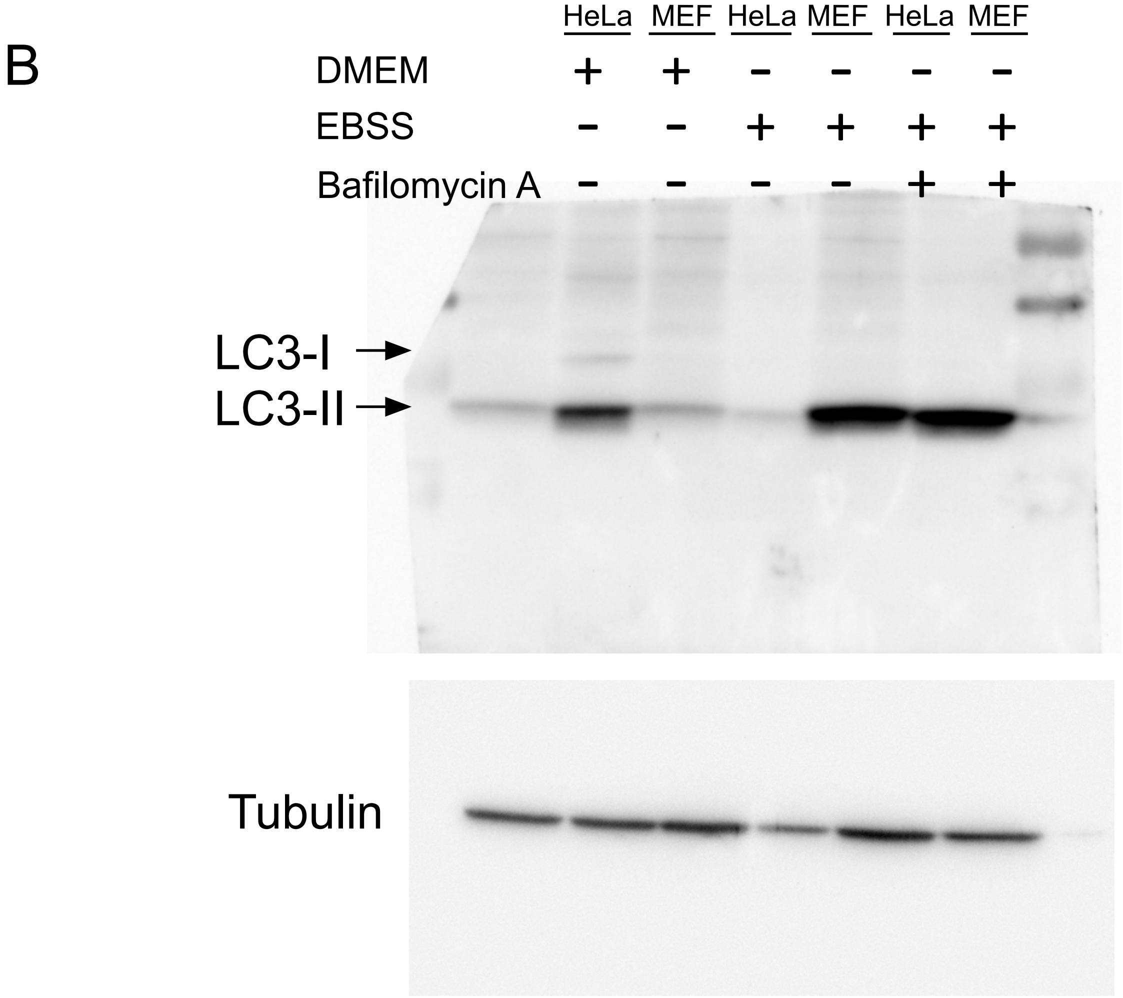

Supplement: S14 Fig — (PDF) [file pone.0230156.s014.pdf]
